# Machine learning predicts clinically significant health related quality of life improvement after sensorimotor rehabilitation interventions in chronic stroke

**DOI:** 10.1038/s41598-022-14986-1

**Published:** 2022-07-04

**Authors:** Wan-Wen Liao, Yu-Wei Hsieh, Tsong-Hai Lee, Chia-ling Chen, Ching-yi Wu

**Affiliations:** 1 Department of Gerontological Health Care, National Taipei University of Nursing and Health Sciences, Taipei, Taiwan; 2 Department of Occupational Therapy and Graduate Institute of Behavioral Sciences, College of Medicine, Chang Gung University, No. 259, Wenhua 1st Rd., Taoyuan, Taiwan; 3 Healthy Aging Research Center, Chang Gung University, No. 259, Wenhua 1st Rd., Taoyuan, Taiwan; 4 Department of Physical Medicine and Rehabilitation, Chang Gung Memorial Hospital at Linkou, Taoyuan, Taiwan; 5 Department of Neurology, Chang Gung Memorial Hospital at Linkou, Taoyuan, Taiwan; 6 College of Medicine, Chang Gung University, Taoyuan, Taiwan; 7 Graduate Institute of Early Intervention, College of Medicine, Chang Gung University, Taoyuan, Taiwan

**Keywords:** Stroke, Rehabilitation

## Abstract

Health related quality of life (HRQOL) reflects individuals perceived of wellness in health domains and is often deteriorated after stroke. Precise prediction of HRQOL changes after rehabilitation interventions is critical for optimizing stroke rehabilitation efficiency and efficacy. Machine learning (ML) has become a promising outcome prediction approach because of its high accuracy and easiness to use. Incorporating ML models into rehabilitation practice may facilitate efficient and accurate clinical decision making. Therefore, this study aimed to determine if ML algorithms could accurately predict clinically significant HRQOL improvements after stroke sensorimotor rehabilitation interventions and identify important predictors. Five ML algorithms including the random forest (RF), k-nearest neighbors (KNN), artificial neural network, support vector machine and logistic regression were used. Datasets from 132 people with chronic stroke were included. The Stroke Impact Scale was used for assessing multi-dimensional and global self-perceived HRQOL. Potential predictors included personal characteristics and baseline cognitive/motor/sensory/functional/HRQOL attributes. Data were divided into training and test sets. Tenfold cross-validation procedure with the training data set was used for developing models. The test set was used for determining model performance. Results revealed that RF was effective at predicting multidimensional HRQOL (accuracy: 85%; area under the receiver operating characteristic curve, AUC-ROC: 0.86) and global perceived recovery (accuracy: 80%; AUC-ROC: 0.75), and KNN was effective at predicting global perceived recovery (accuracy: 82.5%; AUC-ROC: 0.76). Age/gender, baseline HRQOL, wrist/hand muscle function, arm movement efficiency and sensory function were identified as crucial predictors. Our study indicated that RF and KNN outperformed the other three models on predicting HRQOL recovery after sensorimotor rehabilitation in stroke patients and could be considered for future clinical application.

## Introduction

Health related quality of life (HRQOL) refers to the way an individual feels and reacts to his/her health status affected by medical conditions^1^. Compared to quality of life that covers all aspects of well-beings of human life, HRQOL focuses more on well-beings related to health domains such as physical, functional and mental health and it has been regarded as an important outcome of treatments^1,2^.

Stroke remains a leading cause of long-term disability^3^. It has a wide-ranging impact not only on physical and daily function but also on HRQOL^4^. Most patients still suffered from deteriorated HRQOL even in the chronic phase of stroke, which makes HRQOL an crucial target for stroke rehabilitation^4,5^. To improve patients’ HRQOL, healthcare professionals have to provide rehabilitation interventions that are most effective for each patient based on his/her responses to that rehabilitation therapy. Building accurate prediction models for forecasting patients’ HRQOL improvements after rehabilitation interventions and identifying predictors relevant for HRQOL improvements in stroke patients are thus imperative for providing insights to healthcare professionals on making accurate clinical decision.

Machine learning (ML) has become a popular prediction analytic approach. Machine learning uses automatic computerized algorithms to discover patterns in the data and builds prediction models to forecast future events. Machine learning is particularly suitable for predicting health outcomes because it can process large volumes of data, analyze the complex relationship between various different features/variables and easily incorporate new variables into prediction models without re-adjusting the preprogrammed rules^6^. In addition, the feature selection procedure can be incorporated into machine learning procedures to help identify important predictors^7^. These advantages make machine learning a potentially ideal tool for realizing accurate outcome prediction in patient populations.

In stroke, machine learning has been primarily used for predicting motor and activities of daily living (ADL) recovery and has achieved an overall positive result^8–12^. However, to our knowledge, only one study to date has applied machine learning algorithms in predicting stroke-specific HRQOL recovery^13^. In that study, the authors incorporated six demographic factors into machine learning models and built a preliminary system to forecast HRQOL changes of chronic stroke patients. Small prediction errors (i.e., the root mean square errors) were found between the data derived from the prediction model and the actual data collected from the patient, suggesting that machine learning might be feasible for predicting HRQOL changes in chronic stroke patients^13^.

Despite this positive evidence, the previous study only included demographic attributes into the machine learning prediction model^13^; nevertheless, HRQOL has been shown to be affected by factors across multiple domains including demographic as well as health-related domains such as physical and functional domains^4,5,14^. Including only demographic attributes in the machine learning model may not be sufficient for optimizing prediction accuracy. In addition, the previous study only examined prediction errors (e.g., the mean squared error) of the machine learning model^13^. Important clinical performance metrics such as prediction accuracy and the ability of machine learning models to distinguish between responders and non-responders to rehabilitation interventions remain largely unexplored^15^. A comprehensive examination of machine learning prediction performance along with factors across health domains is required for determining the efficacy of machine learning on predicting HRQOL recovery of stroke patients after rehabilitation interventions.

Stroke sensorimotor rehabilitation interventions including the robot-assisted therapy (RT), mirror therapy (MT) and transcranial direct current stimulation (tDCS) have become popular approaches for improving stroke recovery in the recent decade. These three approaches (i.e., RT, MT and tDCS) use modern equipment/modalities (e.g., robotic arms, mirror boxes and electrical stimulators) to modulate peripheral and/or central sensorimotor systems (e.g., visuomotor and sensorimotor systems and cortical areas) to augment stroke recovery^16–18^. Several studies have demonstrated that these three sensorimotor interventions (i.e., RT, MT and tDCS) not only facilitated functional recovery but also improved participation and HRQOL in stroke patients^19–25^. The rationale of why these three sensorimotor interventions (i.e., RT, MT and tDCS) could improve HRQOL is that these interventions could reduce arm/hand impairment, restore arm/hand function, which would allow stroke patients to participate in daily activities and accomplish essential daily tasks^19–25^. Most daily tasks such as bathing, dressing, dining and grocery shopping all involve use of the arm/hand to manipulate objects to accomplish tasks. Good arm/hand function would lead to successful participation in daily tasks and subsequently may increase stroke patients’ subjective feeling of well-beings and satisfaction toward daily life^19–25^. Thus, these three interventions (i.e., RT, MT and tDCS) may have potentials to be incorporated into current clinical practice to facilitate not only functional recovery but also HRQOL in stroke patients. Machine learning may be a potentially useful tool for predicting HRQOL changes after these three interventions, which may help identify responders to these three interventions and facilitate clinical application^6,7^.

Therefore, the purpose of this study was to determine the performance of machine learning algorithms on predicting clinically significant HRQOL improvements of chronic stroke patients after stroke sensorimotor rehabilitation interventions including the RT, MT and tDCS. We examined the performance of five commonly used machine learning algorithms and identified important predictors for building machine learning prediction models.

## Methods

### Study design

This study was an observational cohort study that used secondary analysis of data from our previous randomized controlled or cluster-controlled trials and ongoing projects^24,26–28^. Data screening was done by three investigators (Liao WW, Wu CY and Hsieh YW). The three investigators determined the eligibility and completeness of the data. Patients that completed the interventions and outcome measurements at pre- and post-intervention were included for analysis.

### Participants

One hundred and thirty-two chronic stroke patients (N = 132) were included. Participants were recruited from three hospitals in the northern part of Taiwan. Table 1 outlines the characteristics of participants. The inclusion criteria were (1) a first-ever unilateral ischemic or hemorrhagic stroke, (2) more than 6 months post stroke, (3) Fugl-Meyer assessment scale of upper extremity (FMA) scores between 18 and 60, suggesting mild to moderate arm hemiparesis^29^, (4) no excessive spasticity in upper limb joints (Modified Ashworth Scale, MAS ≤ 3)^30^, (5) ability to follow study instructions (Mini-Mental State Examination ≥ 22), and (6) no concomitant neurological disorders (e.g., brain tumor and dementia). The exclusion criteria were (1) participation in any drug or rehabilitation projects/experiments in the past 6 months, (2) had Botulinum toxin injections in the past 3 months, (3) severe vision or visual perception impairments (e.g., neglect and poor visual field) as assessed by the National Institutes of Health Stroke Subscale, and (4) any contradictions to non-invasive brain stimulation (for participants receiving tDCS)^31^. The institutional review boards of participating hospitals including the Linkou Chang Gung Memorial Hospital and Taipei Tzu Chi Hospital approved the trials. All participants provided written informed consents before enrolled into clinical trials. All study procedures were conducted in accordance with the Declaration of Helsinki.

**Table 1:** Clinical characteristics of participants.

Baseline variables	Participants (N = 132)
Age (years)	55.28 ± 11.66
Gender (male/female)	98/34
Side of lesion (right/left)	73/59
Time since stroke (months)	26.89 ± 22.81
Education (years)	11.29 ± 4.68
MOCA	24.94 ± 4.27
FMA total scores	34.75 ± 9.58
MAS mean scores	0.35 ± 0.6
MRC mean scores	3.83 ± 1.26
WMFT-TIME mean time (s)	11.12 ± 5.58
WMFT-FAS mean scores	2.66 ± 0.6
MAL-AOU mean scores	1.27 ± 0.8
MAL-QOM mean scores	0.91 ± 0.71
BBT-paretic side	9.45 ± 11.3
RNSA-tactile	74.71 ± 28.45
RNSA-proprioception	16.74 ± 5.25
RNSA-stereognosis	13.36 ± 8.65
FIM total scores	111.6 ± 9.63
SIS mean score (%)	65.71 ± 9.94
SIS recovery (%)	50.33 ± 16.48

*MOCA* Montreal Cognitive Assessment assessing global cognitive function (total scores:30), *FMA* Fugl-Meyer Assessment Scale of Upper Extremity assessing motor impairment of the upper extremity (total scores:66), *MAS* Modified Ashworth Scale assessing muscle tone level of the upper extremity (item score range:0–4, the MAS mean scores were the average scores of all parts of upper extremity), *MRC* Medical Research Council Scale for muscle strength assessing muscle strength of the upper extremity (item score range:0–5, the MRC mean scores were the average scores of all parts of upper extremity), *WMFT-TIME* Wolf Motor Function Test TIME-representing movement efficiency of the upper extremity (WMFT-TIME mean scores are the average time of all test items, Unit = seconds), *WMFT-FAS* Wolf Motor Function Test-functional ability scale representing motor function of the upper extremity (item score range 0–5; WMFT-FAS mean scores are the average scores of all test items), *MAL AOU* motor activity log-amount of use representing participants’ self-perceived amount of use of the paretic arm (item score range:0–5; MAL-AOU mean scores are the average scores of all test item), *MAL QOM* motor activity log-quality of movement representing participants’ self-perceived quality of paretic arm movements (item score range:0–5; MAL-AOU mean scores are the average scores of all test item), *BBT-Paretic* Box and Block Test-Paretic representing the paretic hand function (total scores = 150 (150 cubes), *RNSA* Revised Nottingham Sensation Assessment assessing the tactile, proprioception, and stereognosis sensation of the paretic side of the body (item score range: 0–2; unable to test = 9), *FIM* functional independence measure assessing participants’ levels of disability (item score range: 1–7; total score:18–126), *SIS* stroke impact scale.

Value is mean ± standard deviation.

### Stroke sensorimotor rehabilitation interventions

All participants received interventions for 1.5 to 2 h per session with a total of approximately 30 h of training across 3 to 4 weeks. The frequency and duration of training were similar to those of most rehabilitation interventions studies^19–25^. Participants received interventions at the hospitals where they were recruited from. The interventions were administered by certified occupational therapists that were properly trained by the senior therapists and the principal investigators (Wu CY). Among these participants, 70 received RT, 32 received MT and 30 received tDCS/MT.

For the RT, participants practiced unilateral paretic movements by using the InMotion robotic systems (the InMotion ARM and InMotion WRIST)^28^. For the MT, participants imagined that the mirror reflection of the non-paretic arm was the paretic arm and performed bilateral movements as simultaneously as possible^26,27^. For the tDCS/MT, participants received 2 mA anodal tDCS on the ipsilesional primary motor cortex for 20 min followed by another 20 min of MT^24^. For all trainings (i.e., RT, MT and tDCS/MT), participants performed an additional 15–30 min of functional task training in each session. Participants were assessed within 1 week before and after interventions by the evaluators that were blinded to the study purpose and treatment allocation of participants.

### Classification of HRQOL improvement

The Stroke Impact Scale (SIS) 3.0 was selected as the major outcome for classifying HRQOL improvements^32^. It is a self-reported questionnaire used for evaluating HRQOL in stroke patients. The reliability and validity of SIS have been well established^33,34^. The SIS consists of two parts. The first part is the main scale of SIS that assesses multidimensional HRQOL including the strength, hand function, ADL/instrumental ADL, mobility, communication, emotion, memory/thinking, and social participation^32^. It has 59 items and each item was scored subjectively by stroke patients based on the difficulty they perceived in that item during the past two weeks. The scores of each domain were transformed to a score out of 100 and the mean scores of all domains were used to represent multidimensional HRQOL of stroke patients^35,36^. The second part is a global rating scale that evaluates stroke patients’ self-perceived HRQOL recovery. The scores range from 0 (no recovery) to 100 (full recovery). Both parts were included in this study to comprehensively represent multi-dimensional HRQOL and global self-perceived HRQOL changes of chronic stroke patients.

To facilitate clinical use of our ML prediction models, the minimal clinical important differences (MCID) were selected as the criterion to classify participants into high and low responders. The MCID is the smallest change in scores that were considered clinically important and meaningful in health status perceived by the patient^37^. Previous studies have set the MCID as 10–15% of total scores in patient populations and received clinically beneficial results^38–40^. As a result, based on the literatures, we defined the MCID as 10% of changes in the scores of main SIS scale and global rating scale. Participants that had SIS change scores greater than or equal to 10 were classified as high responders and participants with SIS change scores less than 10 were classified as low responders to stroke sensorimotor rehabilitation interventions.

### Candidate predictors

We selected thirty-two potential predictors based on stroke HRQOL literatures and the International Classification of Functioning, Disability and Health (ICF) framework to include “Body function and structures”, “Activity” and “Participation” attributes^41–43^. These predictors included (1) personal characteristic attributes: age, gender, education, time since stroke and side of hemiplegia, (2) baseline cognitive and motor function attributes: The Montreal Cognitive Assessment (MOCA) scores^44^, FMA proximal/distal/total scores^45^, Wolf Motor Function Test (time and functional ability scale) scores^46^, Medical Research Council (MRC) muscle strength scale scores (paretic shoulder, elbow, wrist, and finger muscle strength scores and MRC mean scores)^47^, MAS scores (paretic shoulder, elbow, forearm, wrist, finger scores and MAS mean scores)^47^, Motor Activity Log (MAL) amount of use (AOU) and quality of movement (QOM) scores^48^ and Box and Block test scores^49^, (3) baseline sensory function attributes: the revised Nottingham Sensory Assessment (RNSA) scores (paretic side tactile sensation, proprioception and stereognosis)^50^, (4) baseline ADL and instrumental ADL attributes: Functional Independence Measure (FIM) scores^51^ and the Nottingham Extended Activities of Daily Living (NEADL) scale scores^52^, and (5) baseline HRQOL attributes: the SIS mean scores and global rating scores^32^. These attributes are commonly used in research and clinical settings to represent the motor/sensory impairment, functional ability and participation of stroke patients, and therefore were selected as potential predictors^41–43^.

### Machine learning algorithms

Five ML algorithms, which were the random forest (RF), k-nearest neighbors (KNN), artificial neural network (ANN), support vector machine (SVM), and logistic regression (LG) were used for developing prediction models. The RF uses the ensemble learning method for outcome prediction. It combines the results of multiple decision trees and generates a final overall result to augment prediction accuracy^53^. It is a flexible method that can be used in categorical and continuous data. In addition, the RF has a low probability of overfitting and is therefore suitable for use in the clinical setting^53^. The KNN is a distance-based method. It predicts that similar objects would exist in close proximity. As a result, it labels the class of the target based on the majority of classes of its surrounding k neighbors^54^. The KNN classification pattern is similar to the clinical decision-making process made by the clinicians/therapists, where similar treatments would be prescribed to patients with similar responses and characteristics^55^. The ANN is inspired by the neurological network of the brain^56^. It consists of several neurons/nodes in layers including the input, hidden and output layers. The input layer receives the data, and transfers it to the hidden layer, where the computations (e.g. the activation function) are primarily taken places. After the computations are done, the hidden layer generates the final output to the output layer and finishes the prediction model. The ANN can process complex health informatics data and is therefore a potentially useful tool for outcome prediction in stroke patients^57^. The feedforward back propagation method was used in this study. The SVM uses the binary classification technique, where it projects data onto a high-dimensional plane by using the kernel function first, and then finds the maximum-margin hyperplane that best separates data into two classes^58^. The SVM is efficient in high dimensional planes and suitable for modeling complicated medical data. The LG is a binary classification technique that uses the logistic sigmoid function to predict the probability of observed data that would belong to one of the two possible classes^59^. It is a commonly used algorithm for building prediction models in stroke patients.

These 5 ML algorithms were selected because they are widely used modeling techniques for outcome prediction in patients and have been shown to have good prediction performance^6^.

### Feature selection procedure

The feature selection procedure was performed to remove redundant attributes and identify the essential ones for prediction accuracy^7^. A popular feature selection method called “information gain ratio” was implemented^11^. This feature selection method evaluates the influence (i.e., the information gain) of each attribute to the output classes (i.e., the SIS classes) using the ranker search method^60–62^. A higher gain ratio of the attribute indicates a greater contribution of this attribute to prediction accuracy^62,63^. In this study, attributes with gain ratio greater than zero were used for developing ML prediction models.

### Model development and testing

**Figure 1:**
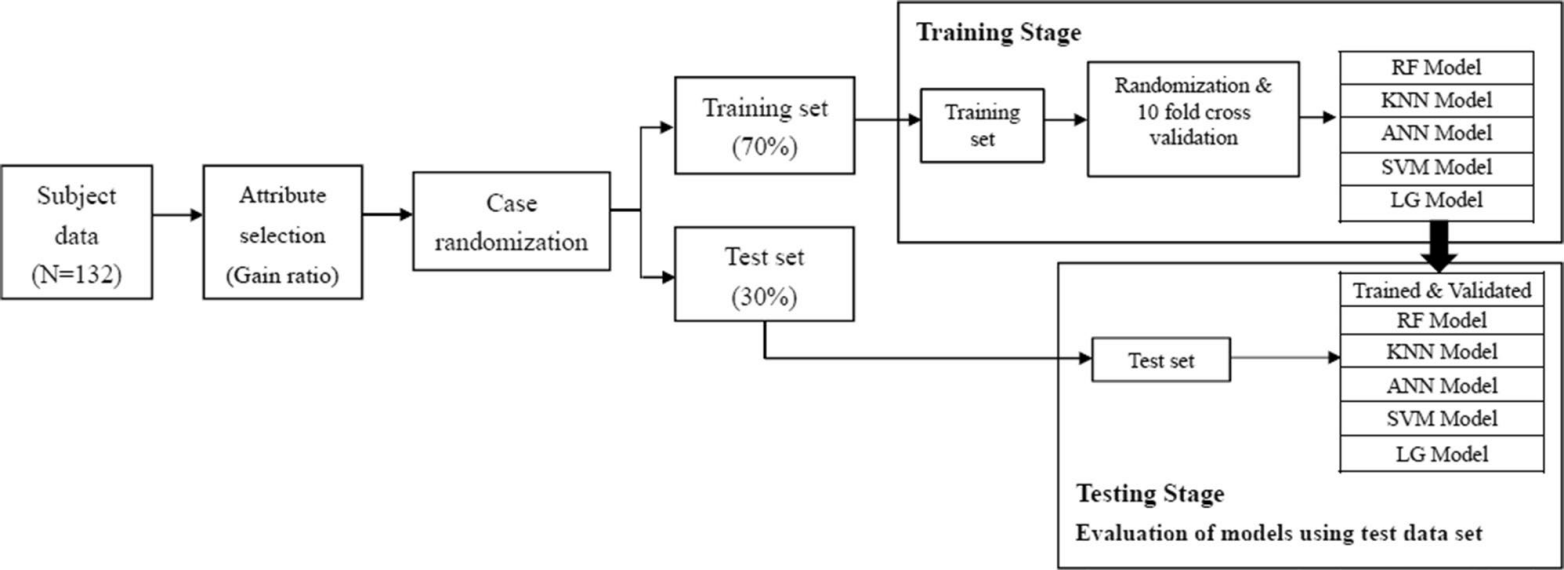
The flow chart of model development and validation process. Subject data were randomized into a training set and a test set. The training set was 70% of the data and the test set was 30% of the data. For the training data set, the tenfold cross validation procedure was used to train and build 5 machine learning models (i.e., the RF, KNN, ANN, SVM and LG) in which the data was randomly split into 10 groups (9 groups for training and 1 group for validation). The tenfold cross validation process repeated until all 10 groups of data were trained and validated. The tenfold cross validation process was performed for all 5 machine learning models. After the 5 models were built, the test data set was entered into the 5 models to determine the model performance.

Figure 1 shows the model development and testing process. Data were randomized and divided into a training data set (70%) and a test data set (30%)^64^. The training data set was used for training and developing the model. The test data set was used for final evaluation of model performance. The tenfold cross validation procedure was performed to train the models^65^. During the tenfold cross validation process, the training data set was split into 10 groups, where 9 of them were used for training the model while the remaining one was used for validating the model. This process was repeated until all groups of data had been trained and validated. After the model was built, the testing data set was entered into the model to assess the model performance.

The hyper-parameters of the prediction models were determined according to the procedures used in the ML literatures. For the RF model, the numbers of trees to build were 100 and the numbers of features to consider at a node were the first integer less than log2M + 1 (M is the number of inputs)^66^. For the KNN and ANN models, the tenfold cross validation was employed to tune the value of hyper-parameters (i.e., the k value of the KNN; the numbers of hidden neurons in the hidden layer of the ANN)^10^. We found that k = 5 and the numbers of hidden neurons = 3 in one hidden layer (the main SIS scale) and k = 9 and the numbers of hidden neurons = 2 in one hidden layer (the SIS global rating scale) had the best prediction accuracy. As a result, these hyper-parameters were used in the KNN and ANN models. For the SVM model, the polynomial kernel function was employed because it provided the best prediction accuracy^58,59^.

### Model performance metrics

The performance of ML models was evaluated using the standard ML performance metrics including (1) accuracy, (2) recall, (3) precision, (4) F1 scores, and (5) area under the receiver operating characteristic curve (AUC-ROC)^15^. Accuracy is an overall index of prediction performance. Accuracy was computed as the sum of true positive (TP) and true negative (TN) divided by the sum of TP, TN, false positive (FP) and false negative (FN). Recall is the ratio of participants that were correctly identified as positive by the model to those whom were actually positive. Recall was computed as the TP divided by the sum of TP and FN. Precision is the ratio of participants that were correctly identified as positive by the model to those were labelled as positive by the model. Precision was calculated as TP divided by the sum of TP and FP. F1 scores is a combined index of precision and recall. It was calculated as the harmonic mean of precision and recall. The AUC-ROC is the ratio of area under the ROC curve to the total area. It represents the ability of the model to distinguish between classes.

### Statistical analysis

The continuous variables were standardized and the categorical variables were coded before developing the ML models. The Waikato Environment for Knowledge Analysis (Weka) 3.8.3 developed by the University of Waikato, New Zeeland was employed for model development, training and testing^67^. The Weka has been extensively used for constructing ML prediction models in various fields and in different patient populations in the medical field^11,68,69^.

## Results

Five most important attributes were identified by the feature selection procedure for the SIS HRQOL main scale, which were the baseline SIS mean scores (gain ratio = 0.1), baseline MRC finger metacarpophalangeal (MP) extensors (gain ratio = 0.15) and flexors (gain ratio = 0.06) scores, baseline MAS wrist flexors (gain ratio = 0.1) scores and baseline WMFT time (gain ratio = 0.14). The gain ratio of the other 31 attributes was 0. As a result, these 5 attributes were used for developing the SIS multidimensional HRQOL prediction model.

Four most important attributes were identified by the feature selection procedure for the SIS global rating scale, including age (gain ratio = 0.16), gender (gain ratio = 0.06), baseline RNSA stereognosis (gain ratio = 0.1) and proprioception (gain ratio = 0.07) scores. The gain ratio of the other 32 attributes was 0. Therefore, these 4 attributes were used for developing the stroke global self-perceived HRQOL recovery prediction model.

Table 2 summarizes the performance metrics of the five ML models. For the SIS multidimensional HRQOL scale, the prediction performance was the best in the RF model. The accuracy of the RF model was 85%, precision was 0.88, recall was 0.85 the F1 scores were 0.85 and the AUC-ROC was 0.86. The prediction performance was similar between the other 4 models (KNN, ANN, SVM and LG). The accuracy ranged from 72 to 75%, the precision was from 0.73 to 0.77, the recall was from 0.73 to 0.75, the F1 scores were from 0.72 to 0.75 and the AUC-ROC was from 0.71 to 0.87.

**Table 2:** Performance metrics of SIS prediction models.

Model	Accuracy (%)	Precision	Recall	F1 scores	AUC-ROC
**SIS main scale**					
RF	85	0.88	0.85	0.85	0.86
KNN	75	0.76	0.75	0.75	0.8
ANN	75	0.77	0.75	0.74	0.87
SVM	72	0.73	0.73	0.73	0.71
LG	72.82	0.73	0.73	0.72	0.77
**SIS global rating scale**					
RF	80	0.78	0.8	0.78	0.75
KNN	82.5	0.82	0.83	0.81	0.76
ANN	77.5	0.77	0.78	0.77	0.75
SVM	77.5	0.77	0.78	0.77	0.68
LG	77.5	0.78	0.78	0.78	0.75

*SIS* Stroke Impact Scale, *RF* random forest, *KNN* k-nearest neighbors, *ANN* artificial neural network, *SVM* support vector machine, *LG* logistic regression, *AUC-ROC* area under the receiver operating characteristic curve.

For the SIS global rating scale, the prediction performance was the best and similar between the RF and KNN model. The accuracy of the RF model was 80%, the precision was 0.78, the recall was 0.8, the F1 scores were 0.78 and the AUC-ROC was 0.75. The accuracy of KNN model was 82.5%, the precision was 0.82, the recall was 0.83, the F1 scores were 0.81 and the ACU-ROC was 0.76. The prediction performance was similar between the other three models (ANN, SVM and LG). The accuracy was all 77.5%, the precision was from 0.77 to 0.78, the recall was all 0.78, the F1 scores were from 0.77 to 0.78 and the AUC-ROC was from 0.68 to 0.75.

## Discussion

Our results demonstrated that machine learning could accurately predict HRQOL improvements after stroke sensorimotor rehabilitation interventions in chronic stroke patients. In particular, the RF and the KNN models had better performance than the other three algorithms (i.e., ANN, SVM and LG). The RF model had 85% accuracy on predicting multidimensional HRQOL changes and 80% accuracy on forecasting global self-perceived recovery. It could also accurately distinguish between high and low responders on the multidimensional HRQOL outcome with 86% chances and on the global self-perceived recovery with 75% chances. The KNN model had good prediction performance on global self-perceived recovery only, where it had 82.5% prediction accuracy and could distinguish between high and low responders with 76% chances. Furthermore, we identified important attributes for predicting multidimensional HRQOL improvements, which were the baseline HRQOL, baseline paretic finger muscle strength, wrist muscle tone and arm movement efficiency, and also key attributes for forecasting global self-perceived recovery including age, gender, baseline hand stereognosis and limb proprioception.

To our knowledge, this study is the first to comprehensively evaluate the performance of ML algorithms on predicting HRQOL improvements after stroke sensorimotor rehabilitation interventions in stroke patients. In addition, we identified the two more effective algorithms, which were the RF and KNN among 5 commonly used ML algorithms. The RF algorithm had good prediction performance on both multidimensional HRQOL and global self-perceived recovery. This good prediction performance may be contributed by the unique ensemble method that it employed in the modeling process. During the ensemble modeling process, the RF creates as many base models (i.e., the decision trees) as many as possible and combines these base models into a final one^66^. Therefore, instead of creating one model and hoping this model would be the best, the RF takes a myriad of multiple models into account to optimize its prediction accuracy. Furthermore, these base models (i.e., the decision trees) are designed to be uncorrelated with each other, thus reducing the probability of overfitting^66^. Our finding of the superior performance of RF was in line with several previous studies demonstrating that ML algorithms with ensemble methods such as the RF and the Adaboost algorithms outperformed other types of ML algorithms, for example the decision trees or LG^63,70–73^. Future studies could examine the performance of ML algorithms with different types of ensemble methods such as the boosting, bagging and stacking on HRQOL outcome prediction to determine the best one for use in stroke patients.

To our surprise, the KNN algorithm has similar and slightly better prediction performance than the RF algorithm on predicting global self-perceived HRQOL recovery in chronic stroke patients. Compared to the RF, the KNN is a simpler and more straightforward model because it is a distance-based method and does not involve ensemble learning processes^54,55^. Our study revealed that despite its simplicity, the KNN may be as powerful as the RF model when predicting a single-item outcome such as the SIS global self-perceived HRQOL recovery scale in stroke patients. In contrast, the KNN may not be the best algorithm for processing multidimensional health outcome data due to its weaker prediction performance on multidimensional than single item SIS HRQOL outcomes. As a result, we recommend using the KNN model for predicting simple HRQOL outcome changes in chronic stroke patients. Future studies could compare and contrast the performance of KNN on single domain and multidimensional health outcome data to validate findings of this study.

Our study showed that 5 attributes related to initial HRQOL, muscle function (the muscle strength and muscle tone) and movement efficiency were important predictors for forecasting multidimensional HRQOL improvements after sensorimotor rehabilitation interventions. Indeed, studies have found that baseline HRQOL was associated with HRQOL restoration in stroke patients^74,75^. It is thus not surprising to find baseline SIS scores important for HRQOL improvements after interventions. In addition, studies have shown that paretic arm/hand muscle strength and muscle tone significantly affected functional recovery after stroke^43,76,77^. The movement efficiency of the paretic arm was also associated with HRQOL recovery post stroke^5^. Similarly, in the present study, we found that muscle function (i.e., baseline MRC finger MP extensors/flexors, MAS wrist flexors) and the movement efficiency (i.e., baseline WMFT time scores) of the arm associated with prediction of HRQOL improvements. However, compared to previous studies, we further identified the most important components, which were the muscle function of “the wrist/hand” and the movement efficiency of “the whole arm”. These components are highly involved in daily routines. For example, stroke patients have to be able to appropriately open/close their hands, grasp/release objects and move their arms efficiently in time to perform most essential daily tasks such as dressing, bathing and cooking. The inability to accomplish essential daily tasks may result in a source of distress and consequently affect participation and life satisfaction after stroke^78^. Therefore, even though muscle function and movement efficiency are commonly regarded as the basic level (i.e., body functions and structures) of the ICF model, they could substantially affect the multi-dimensional HRQOL recovery after stroke sensorimotor interventions and therefore should be considered when assigning these interventions to stroke patients.

In addition, our study also found another four attributes imperative for forecasting global self-perceived HRQOL recovery, which were age, gender and the paretic hand stereognosis and limb proprioception. Age and gender have been demonstrated to be associated with HRQOL recovery post stroke in previous studies^5,79^. Our results support theses previous findings and further suggest that additional somatosensory impairments, especially the sensory components including the hand stereognosis and limb proprioception are also important for predicting global self-perceived HRQOL recovery. Indeed, reduced sensation has been found to be related to slower recovery, decreased motor function (e.g., motor control and activity) and lesser rehabilitation outcomes^43,80,81^. Sensory deficits, such as impaired hand stereognosis and limb proprioception may cause difficulties in sensing arm/hand position and recognizing objects in the hand and hard to control arm/hand movements. This may result in decreased confidence in using the arm/hand in daily activities, fear of safety, thus affecting stroke patients’ participation and satisfaction in daily life^80–82^. Our results were also in line with one previous study that found paretic limb proprioception associated with reduced HRQOL and increased feeling of social isolation in stroke patients^14^. Nonetheless, most studies did not assess the contributions of sensory impairments on HRQOL outcome prediction after rehabilitation interventions in stroke patients^83^. Our study suggested that sensory impairment may contribute to global self-perceived HRQOL recovery post stroke intervention and could be considered in the data collection and HRQOL model building process.

Taken together, we found that attributes in the three major categories: the personal characteristics (i.e., age and gender), initial life satisfaction (baseline SIS mean scores) and arm/hand sensorimotor components (i.e., muscle function, movement efficiency, hand stereognosis and limb proprioception) were important predictors for HRQOL restoration after stroke sensorimotor rehabilitation. Based on our findings, healthcare professionals could at least assess these attributes in the three categories before assigning the three sensorimotor interventions to stroke patients. In addition, these attributes may have potential to be used as indicators to determine which stroke patient may be suitable for receiving stroke sensorimotor rehabilitation interventions to improve HRQOL. This may help to improve clinical rehabilitation efficacy and potentially save workload in the hospitals/clinics.

### Study limitations

Six limitations should be considered. First, our HRQOL outcome prediction was focused on stroke sensorimotor rehabilitation interventions that share similar rehabilitation principles. Future studies could examine whether the identified predictors, such as the sensorimotor components could generalize to HRQOL outcome prediction in other types of rehabilitation interventions. Second, our predictions were based on changes immediately after interventions. Future studies could investigate the prediction performance of ML algorithms in the follow-up period. This will help identify patients that could retain improvements after sensorimotor interventions. Third, we examined 5 commonly used ML algorithms. Future studies could evaluate the performance of other types of ML or deep learning algorithms such as those with ensemble methods (e.g., the Adaboost)^84^ or the deep learning algorithms (e.g., the deep neural network)^85^ and compare the results to our findings to determine the optimal method for predicting HRQOL restoration after rehabilitation interventions in stroke patients. Fourth, this study included thirty-two potential predictors based on the results of previous prediction model studies. Nevertheless, some stroke-related factors were not included, for example, the size/severity of lesion, due to the incapability to retrieve those data from some of our patients. Future studies could examine if these stroke related factors would also be important predictors for forecasting HRQOL recovery in chronic stroke patients, in addition the predictors identified in this study. Fifth, we used SIS to assess multi-dimensional HRQOL and self-perceived global HRQOL changes in stroke patients. Although SIS is a widely used HRQOL assessment in stroke rehabilitation field, it is an ordinal questionnaire that may still have measurement errors. In addition, the SIS may not cover all aspects of HRQOL related factors such as environmental factors (e.g., the context and time), personal factors (e.g., personality) and social indicators (e.g., economic status). We encourage future researchers to include the above factors into machine learning prediction models and examine whether inclusion of these additional factors would optimize prediction accuracy on HRQOL. Sixth, the machine learning models built in this study were preliminary models that focused on predicting HRQOL recovery. Therefore, these models should not be used for excluding patients from receiving the three stroke sensorimotor interventions at this moment. Future studies could include more participants and develop machine learning models that comprehensively predict motor, functional and HRQOL recovery in stroke patients.

## Conclusion

Machine learning may predict clinically significant HRQOL improvements after stroke sensorimotor rehabilitation interventions in chronic stroke patients. In particular, the RF and the KNN algorithms may be more effective than the other 3 ML algorithms (DT, LG and SVM) and could be considered for use in clinical settings. We suggest including at least three categories of predictors, which are age/gender, initial HRQOL and sensorimotor components including sensory function, muscle function and movement time into the ML HRQOL prediction model for optimizing prediction accuracy. Future studies with a different sample of stroke patients are warranted to validate our findings and improve model generalizability.

## Data Availability

The datasets used and/or analyzed during the current study are available from the corresponding author on reasonable request.
